# Perceptions and Attitudes of People With Cancer and Diabetes Towards Patient Guidelines: A Mixed Methods Study

**DOI:** 10.1111/hex.70164

**Published:** 2025-01-28

**Authors:** Lijiao Yan, Ning Liang, Zeyu Yu, Loraine Cook, Sarah E. Scott, Karen Graham, Xing Liao, Haili Zhang, Jiale Hu, Nannan Shi, Jianping Liu

**Affiliations:** ^1^ Institute of Basic Research in Clinical Medicine, China Academy of Chinese Medical Sciences Beijing China; ^2^ Centre for Evidence‐Based Chinese Medicine, Beijing University of Chinese Medicine Beijing China; ^3^ School of Education, The University of the West Indies Kingston Jamaica; ^4^ Healthcare Improvement Scotland Edinburgh Scotland UK; ^5^ National Institute for Health and Care Excellence (NICE) Manchester UK; ^6^ Department of Nurse Anesthesia Virginia Commonwealth University Richmond Virginia USA

**Keywords:** attitudes, awareness, clinical practice guidelines, mixed methods, patient guideline, survey

## Abstract

**Objective:**

To explore patients' perceptions and attitudes towards patient guidelines (PGs) and to identify specific factors related to PG content, design, presentation, and management that may influence patients' use or adoption of PGs.

**Methods:**

An exploratory sequential mixed‐methods design was employed. Initial semi‐structured interviews were conducted with a diverse group of individuals, including people with diabetes or oncology, and clinicians. These interviews were analysed through directed content analysis. Findings from the qualitative study were used to develop a questionnaire. The questionnaire was circulated to patients with diabetes and cancer and asked them to report their awareness, attitudes, and the PG‐related factors influencing their use and adoption of PGs.

**Results:**

In total, 25 participants were interviewed qualitatively, and 400 participated in the quantitative survey. Analysis of interviews yielded three themes: perception of PGs, attitude towards PGs, and key PG attributes influencing patients' use or adoption of PG. Qualitative findings indicated limited awareness of PGs, supported quantitatively by only 26.5% of patients being aware of PGs. Attitudes varied, with 73.0% expressing an overall positive attitude towards PG, but only 17.3% preferred PGs for evidence‐based answers and 32.3% favoured them for decision support, citing concerns that general recommendations may not meet individual needs. Participants suggested tailoring recommendations based on subgroups considering age, comorbidity, and weight, explaining why treatments work or don't work in different populations. Eight PG attributes influencing their use or adoption were found: accessibility, identifiability, attractiveness, credibility, usability, timeliness, relevance and simplicity. Lack of credibility was the most frequently mentioned hindrance, with 34.8% identifying unverifiable information as a barrier. Further qualitative and quantitative analyses revealed that medical staff were trusted sources for conveying PGs to patients.

**Conclusions and Practice Implications:**

This study underscores the necessity for PGs to acknowledge individual differences and provide recommendations that are more tailored considering age, comorbidities, weight, and other factors influencing decision‐making, ensuring that they address patients' specific needs and support informed decision‐making. Additionally, there was a significant need to improve the dissemination of PGs, using medical staff as key channels to improve patients' use or adoption of PGs.

**Patient or Public Contribution:**

In this study, patients were actively involved in several stages. During the development of the interview guide, feedback from two patients, alongside one patient guidelines (PGs) developer and three clinicians, was incorporated to ensure the guide's relevance and comprehensiveness. Patients' insights were integral to refining the interview questions, ensuring they were appropriate and effective. Additionally, the survey questionnaire was pre‐tested among 20 patients using the Think‐Aloud method, which led to significant revisions for better comprehension and response quality. These steps highlight the essential role of patients in shaping the data collection instruments and enhancing the overall quality and relevance of the study.

**Abbreviations**
COREQ: Consolidated Criteria for Reporting Qualitative Research
CPGs: clinical practice guidelines
GIN: Guidelines International Network
GRAMMS: Good Reporting of A Mixed Methods Study
PDAs: patient decision aids
PEMs: patient education materials
PGs: patient guidelines
PVG: patient version of guidelines
SIGN: Scottish Intercollegiate Guideline Network
TCM: Traditional Chinese Medicine

## 1 Introduction

As healthcare systems worldwide move towards more patient‐centred approaches, clinical practice guidelines (CPGs) and their derivative products such as the patient version of guidelines (PVGs) [1] for public use have emerged as essential tools to enhance patient understanding and involvement in medical decision‐making. CPGs are statements aimed at assisting clinicians and patients in making decisions about appropriate healthcare for specific clinical circumstances [2]. These CPGs are developed through a rigorous process, including systematic evidence review, quality assessment, and strength‐based recommendation formulation. The strength of recommendations is determined by guideline panels based on the balance of benefits and harms, evidence certainty, and patient values [3]. Strong recommendations suggest an intervention is suitable for most patients, while weak recommendations indicate decisions may vary and require individualization by doctors based on patient needs and preferences [4]. The development of CPGs involves a panel of multidisciplinary experts and representatives from stakeholders, including patients and their caregivers. This comprehensive approach ensures that CPGs offer reliable, up‐to‐date recommendations to optimize patient care [5]. While CPGs are primarily designed for clinicians, PVGs serve as patient‐directed knowledge tool to ‘translate’ CPG recommendations and their underlying rationales for the broader public, making them comprehensible for a wider audience [1].

Because many different patient‐directed knowledge tools exist and their purpose, and scope are often not clearly defined and distinguishable from one another, this can be confusing for both users and PVG developers [6]. To clarify the distinctions between these tools, it is important to highlight the specific focus on PVGs. The following key types of patient‐directed knowledge tools can be differentiated based on their purposes in patient education and decision‐making: patient education materials (PEMs), PVGs, and patient decision aids (PDAs). A detailed comparison of these tools, including their roles and functions, is provided in the attached table (see Appendix, Table A1). These distinctions are based on the framework developed by Dreesens et al. [7]. In China, PVGs differ from international practices in their development process. International PVGs are typically translated versions of CPGs [1], whereas Chinese PVGs can include both translated versions of CPGs and those that are adapted from existing CPGs or created from scratch to better address patient‐centred concerns [8]. These locally developed versions use non‐specialist language to improve comprehension and are often referred to as patient‐directed guidelines. For the purposes of this study, we will use the term patient guidelines (PGs) to refer to Chinese PVGs; their goals and functions are consistent with those of international PVGs, with the primary distinction lying in their development methods.

PGs help to foster a trustworthy patient‐clinician relationship by providing understanding about how, based on the evidence, clinicians should treat a condition. In turn, people may feel reassured and confident in their care [1]. However, the success of PGs largely depends on patients' awareness, understanding, and trust in these tools. Understanding patients' attitudes and perceptions towards PGs is critical, as they shape the extent to which patients engage with PGs and make informed decisions. If patients are unaware of these resources, or if they perceive them as too complicated, irrelevant or untrustworthy, PGs' potential to improve patient outcomes is diminished. Furthermore, identifying specific factors related to the content, design, presentation, and management of PG that influence their use and adoption by patients is crucial for optimizing their effectiveness. By understanding which aspects of PGs enhance or hinder their use, we can develop more targeted strategies to improve their clarity, relevance, and credibility from the patient's perspective.

Recent studies provide valuable insights into patients' perceptions and attitudes towards CPG or PGs. For example, research on patient and public perceptions and attitudes towards specific CPGs or PGs has highlighted that there was significant variability in public awareness of such guidelines, ranging from 0% to 79% [9, 10, 11, 12, 13] depending on the population and context surveyed. A systematic review of public attitudes towards CPG found mixed attitudes, with some individuals finding the CPG empowering, while others viewed them as tools for rationing care [14]. A study on user testing of a Scottish Intercollegiate Guideline Network (SIGN) PG revealed that although participants appreciated the accessible design features (such as a clear purpose, friendly tone, and simple language), they were often disappointed by the limited scope of the information provided, particularly regarding treatment options [15]. The Canada Physical Activity Guideline has been criticized for its overly simplistic, cartoon‐like format, which some participants felt undermined its credibility [16]. Additionally, several studies have shown that participants frequently found the terminology unfamiliar and questioned the necessity of presenting evidence quality or certainty in PGs [17, 18].

While efforts were being made to comprehend patients' perceptions and attitudes towards PGs, these findings predominantly derive from Western populations. Whether these insights are universally applicable remains unknown, given the significant variations in health literacy and information needs shaped by socioeconomic conditions [19, 20]. China, with its large and diverse population, presents a unique healthcare environment. Cultural norms that emphasize reliance on physician authority [21] and generally lower levels of patient participation in medical decision‐making [22] distinguish China's healthcare landscape from that of many Western countries. These factors may impact how patients in China perceive and engage with PGs. As such, the conclusions drawn from Western‐based studies may not fully apply to the Chinese context, where a different set of challenges and expectations could shape patient attitudes towards PGs. For instance, the Guidelines International Network (GIN) [1] recommended presenting the level of evidence and strength of recommendation to patients. However, a completed scoping review of PGs in China conducted by BUCM team members [23] revealed that most Chinese PGs either only present the recommendation level [24] or omit such information [25]. Whether this relates to different country populations' characteristics warrants further exploration. As of 20 February 2022, 26 PGs (both ongoing and completed) targeting various aspects of medical care, such as diabetic disease, rheumatoid arthritis, and ischaemic stroke, have been identified in China [23]. Despite this, research on their impact on patients is scarce, leaving a gap in understanding the extent to which PGs contribute to informed choices and shared decision‐making.

Therefore, this study explores patients' perceptions and attitudes towards PGs and the factors influencing their use, using a mixed‐methods approach. We focus on diabetes and cancer, as these are common chronic conditions that affect a large portion of the population [26, 27]. Additionally, the complex treatment options in diabetes and cancer care require patients to be more involved in decision‐making [28, 29], making PGs especially valuable in helping them understand their options. The findings from this study will inform recommendations for developing and implementing PGs in China and similar contexts.

## 2 Materials and Methods

This study used an exploratory sequential mixed methods design, consisting of two phases: qualitative and quantitative. In the first phase, semi‐structured interviews were conducted with patients, clinicians, and nurses to explore perceptions of PGs. The results informed the development of a structured questionnaire used in the second quantitative phase. The study adhered to the Good Reporting of A Mixed Methods Study (GRAMMS) [30] and the Consolidated Criteria for Reporting Qualitative Research (COREQ) [31].

### 2.1 Sample and Setting

The study was conducted across four hospitals in Beijing and Shenzhen. Patients aged 18 or older, diagnosed with high blood glucose or oncology conditions, and clinicians with at least 5 years of experience were included. Purposive sampling ensured representation across key demographic variables.

### 2.2 Data Collection

Qualitative data were collected from May to August 2022 through semi‐structured interviews, either online or face‐to‐face. Quantitative data were collected from August to November 2022 via an interviewer‐administered questionnaire on the WenJuanXing platform.

### 2.3 Data Analysis

Qualitative data were analysed using directed content analysis with NVivo software. Quantitative data were analysed with descriptive statistics, and integrated findings were presented using a weaving approach and joint display [32].

Detailed information about the methodology, including sample selection, data collection instrument, and analysis procedures, is provided in the Appendix.

## 3 Results

### 3.1 Characteristics of Participants

#### 3.1.1 Characteristics of Participants From the Qualitative Interviews

Of the 25 individual qualitative interview participants, 11 were people living with diabetes and 5 were oncology patients, 4 were medical and nursing staff of oncology, 4 were medical and nursing staff of endocrinology and 1 was general practitioner. All participants had junior high school degree, with a mean age of 44.3 years, ranging from 27 to 64 years (see Appendix, Table A2).

#### 3.1.2 Characteristics of Survey Population

Overall, 137 oncology patients and 263 people living with diabetes participated (*N* = 400). There is a balanced numbers of male and female among survey population with age ranging from 19 to 81 years (see Appendix, Table A3).

### 3.2 Result of Qualitative and Quantitative Studies

Our analysis of interviews yielded 3 themes, with 13 sub‐themes. These three themes were perception of PGs, attitude towards PGs and key PG attributes influencing patients' use or adoption of PGs. Both qualitative (see Appendix, Table A4) and quantitative results (see Appendix, Table A5) were reported based on these three themes.

#### 3.2.1 Perception of PGs

This study found that patients and clinicians lacked a clear understanding of what PGs were. Even among those who claimed to be familiar with PGs, their understanding was limited to viewing PGs as general health education materials or medical care guides, disease‐related informational books.

I think if there is a patient guideline, when I am sick, I myself will be able to know what kind of problem I have, how to deal with this problem, which hospital I should go to, how to make appointment with a doctor, which doctor I should see. As many people lack that information, the guideline will be helpful to us.

[Patient C]

This was further illustrated in the quantitative findings: only 26.5% of patients reported having heard of PGs. However, among those, 71.7% regarded PGs as general health education materials or as medical care guides.

#### 3.2.2 Attitudes Towards PGs

1. Attitudes towards PGs as a whole
  Attitudes towards PGs were heterogeneous, some patients and clinicians believed that they would benefit from PGs, as they were professional and scientific, and developed from the perspective of patients. However, some patients indicated PGs were not helpful for them, and they won't seek PGs, information conveyed by healthcare providers is enough. For example,
  Diabetes is all about that little bit of knowledge, what I should know the doctor will surely inform, too much other information can't cure myself, because there will be no new knowledge.
  [Patient B]
  Congruently, 73% of patients expressed willingness to seek health‐related information beyond healthcare providers, including PGs. While 27% indicated they had no interest in seeking information from other sources.
2. Attitudes towards recommendations for patients in the PG
  Regarding the recommendations for patients in the PG, some patients believed that these recommendations were scientifically sound and credible; and that these recommendations would help them make better decisions, especially in the following situations: inadequate communication from doctors, inconsistent solutions from different healthcare providers, a divergence between their ideas and doctors' opinions, discrepancies between their own views and those of their doctors, and inconveniences in addressing certain daily life‐related confusions with doctors.
  For example, with diabetes, one doctor might recommend insulin while another suggests pills. Patients often don't know which option is better. In such cases, a scientific recommendation from a PG could clarify the best approach and help me make a more informed decision.
  [Patient E]
  However, some patients expressed concern that recommendations in the PG might not be applicable to their individual needs. The results of the studies underpinning recommendations were based on a population in other context, which could lead to a lack of suitability for individuals in varying situations. Additionally, patients felt that experts may not fully understand their unique circumstances.
  Nevertheless, the participants expressed a continued willingness to consider the recommended treatment in PGs. The only caveat was that they hoped, the recommendations should be presented based on different subgroups taking account of age, co‐morbidity, weight and explaining why treatments work or don't work in different populations.
  Let's take a closer look at blood sugar levels as an example. In individuals with type 1 diabetes, even with the same food and medication dosage, their post‐meal blood sugar levels can differ. This happens because not everyone within a particular group is identical. There are multiple categories, and each may have distinct factors affecting their blood sugar levels. If patients are provided with guidance on which category they might fall into, it could help them better understand their condition and adjust their management plans accordingly.
  [Patient G]
  This was also confirmed in the quantitative findings. 60.25% of the patients expressed a desire for recommendations from PGs, which were provided by a group of experts from multiple disciplines based on the best available evidence when facing decision‐making problems, but only 17.3% preferred PGs for evidence‐based answers. Furthermore, approximately 181 patients (75.11%) would prefer recommendations tailored to their specific health condition, such as their age, comorbidities, and other personal factors.
3. Attitudes towards the presentation of strength of recommendation
  During the interviews, some participants indicated that the strength of the recommendation in PGs made it more credible and influenced their choices, helping them move beyond basing decisions solely on their preferences.
  Yes, from my personal preference, well, I would choose diet B (the comparison), but if I know the recommendation level of intervention A is strong, that is to say, in terms of scientific evidence and data on the health benefits, intervention B is better for us, then you can't diet according to your own preferences, you know? Because your own preferences don't determine the science.
  [Patient L]
  However, some participants believed that the information was not helpful to them.
  Personally, I think it's pretty much the same. If you make recommendations to us in the guidelines, why do you need to grade it? For example, if the guideline recommends that I (as a person living with diabetes) should run 1,000 meters, but I'm only able to run 600 meters, so it doesn't make sense for me whether the recommendation is strong or weak? I may still have to decide whether I take the advice according to my condition? I don't think it matters whether it's graded strong or not.
  [Patient F]
  Interestingly, when we presented Patient D with a weak recommendation against using a neutropenic diet during tumour treatment, she initially found it confusing. However, once we explained the meaning of ‘weak’ and the rationale behind assigning this level to the recommendation, Patient D gained a better understanding of it and felt more informed and reassured about her choices.
  Yes, it makes sense to give a weak recommendation. As you know, I am adopting a neutropenic diet, I was just thinking that my diet was not in line with the recommendation and I was hesitant if I should do it in future, but after I realized that the guideline gives a weak level to this recommendation, I'm now more confident with my choice (adopting neutropenic diet) before, because everyone has different food tolerances and this recommendation is not necessarily suitable for everyone.
  Regarding the icons (e.g. using thumbs up, tick and question mark symbols recommended in the GIN public toolkit [1] to communicate the strength of recommendation used in the existing PGs, participants found them challenging to understand, or they were easily misinterpreted. For instance, some participants mistakenly associated the strength of the recommendation with the quality of the recommended treatments, leading them to desire only strong recommendations from the PGs. Additionally, there was a misunderstanding among some participants that a strong recommendation indicated that their condition was more severe or required urgent treatment.
  Guideline will certainly give you different strengths of recommendation based on the severity of the disease. If the guideline recommend treatment to you with strong recommendation, this means you are very sick, should take the treatment as soon as possible. It is unlikely to give you strong recommendations for minor ailments, if they do, I wouldn't accept that, because the guideline must be scaring you.
  [Patient H]
  This was further illustrated in the survey, where 69.75% of patients indicated that the strength of recommendation would impact their decision‐making. Specifically, 40.75% of patients said that the recommendation strength would influence their decision to adopt the recommendation, while 29.00% stated that they would only follow strong recommendations. Additionally, 23.00% of patients chose that the strength of the recommendation would not affect their adoption of the recommendation, and they were only concerned with the recommendation itself (see Table 1).
4. Attitudes towards PG in assisting patients in decision‐making.

**Table 1:** Joint display of qualitative with quantitative findings.

Key findings	Qualitative evidence	Quantitative evidence	Interpretation	Finding type
**Perception of PGs**				
Patients and clinicians have limited understanding of PGs, often seeing them as general health guides.	Patients and clinicians have a limited understanding of PGs, commonly described PGs as general health education materials or guides for medical care	Only 26.5% of patients had heard of PGs, and 71.7% of them saw PGs as general health materials	Both methods highlight a limited understanding of PGs, typically regarded as general health information rather than patient‐specific management tools.	Confirmed Findings
**Attitudes towards PGs**				
Attitudes towards PGs are heterogeneous	Some patients and clinicians saw PGs as professional, scientific and patient‐centred, while others felt they wouldn't be useful. Some patient stated that healthcare provider information was sufficient	73% of patients expressed interest in PGs, with 27% showing no interest in seeking health‐related information outside of healthcare providers	The qualitative evidence provides insight into patients' reasons for or against PGs, while the quantitative evidence reflects the level of willingness to engage with them. The data confirme and complement each other by showing both diverse attitudes and overall willingness to seek PGs	Confirmed and expanded findings
Patients desire PG recommendations but prefer personalized, context‐sensitive guidance	Some patients find PG recommendations scientifically credible but want more personalized advice tailored to their condition	60.25% of patients desire expert‐driven PG recommendations for decision‐making, and 75.10% of patients prefer recommendations tailored to their individual needs or conditions	Both sets of findings highlight that patients value personalized recommendations, with the qualitative results reflecting this desire and the quantitative data confirming the extent of this preference	Confirmed Findings
Strength of recommendations influences decision‐making for some patients, while others find it unhelpful	Some participants felt the strength of recommendations helped them make more informed choices, while others felt the strength of recommendations didn't affect their decisions, focusing on individual circumstances. Some participants also misunderstood the recommendation levels, associating them with disease severity or thinking only strong recommendations were valid	69.75% of patients indicated that the strength of recommendation influenced their decision‐making; 40.75% said it would influence adoption, while 29.00% would only follow strong recommendations. 23.00% stated that the strength of recommendation did not affect their decision	The quantitative data supports the qualitative finding that patients have differing views of the strength of recommendations, and the quantitative results elaborate these views, by quantifying the proportions of patients with each view	Confirmed and expanded findings
Patients have varying preferences regarding the use of PGs for decision‐making	Some patients felt that decision‐making should be left to clinicians and preferred clear explanations of treatment options, including benefits and risks. Others expressed a need for tools to support independent decision‐making	32.25% of patients said they needed a PG to assist them in decision‐making independently. 28.75% preferred PGs that support informed decision‐making without requiring independent choices, 25.00% said they don't need a PG and prefer to rely directly on the doctor	The quantitative data supports the qualitative finding that there is diversity of patient preferences regarding the use of PGs for decision‐making. The quantitative evidence expands this diversity by quantifying specific proportions of patients with each preference	Confirmed and expanded findings
**Key attributes of PG influencing patients' use or adoption of PGs**				
Accessibility of PGs influences how easily patients can find and use them	Patients emphasized that ease of access is critical in seeking and using PGs, Patients preferred easily accessible materials, such as those directly provided by doctors, rather than searching for information themselves (e.g., via WeChat) PGs can be distributed by the hospital during the visit, by the community, etc. If the PG is not directly distributed to patients, it should be findable Preference for direct accessibility: PGs that can be easily accessed on mobile devices without needing to search or navigate through other platforms	24.16% (43/178) of patients viewed difficulties in accessing PG or other health information as a barrier for seeking or reading PG or other health information	The quantitative data supports the qualitative finding that accessibility affects patient engagement with PGs, confirming that it is a real barrier, even though the proportion is modest. Additionally, the qualitative findings provide depth by explaining why and how accessibility matters (e.g., patients dislike searching for information themselves), while the quantitative findings extend the qualitative insights by illustrating how prevalent this barrier is within the patient population	Confirmed and expanded findings
Identifiability of PGs helps patients recognize and relate to the content quickly	Patients emphasized the importance of quickly recognizing whether a PG is relevant to them. They suggested that categorizing content or using clear frameworks, such as STAR or SCQA, would help them quickly identify the most pertinent information	21.35% (38/178) patients perceived the initial inability to discern the relevance of the PG or other health information to them (patients) at first glance as a barrier for seeking or reading PG or other health information	Both qualitative and quantitative results show that identifiability affects patient engagement. The qualitative findings explore how categorization and clear structuring (e.g., STAR, SCQA) can aid recognition, while the quantitative results highlight that over one‐fifth of patients face identifiability challenges. This complementary relationship illustrates how qualitative data offers solutions for improving identifiability, while quantitative data reveals the extent of the issue.	Confirmed and expanded findings
Credibility of PGs ensures patients trust the information and recommendations provided	Patients expressed concerns about the credibility of information in PGs, citing an overwhelming amount of misleading information in the media that exaggerates facts for attention. They also value the reputation of the organization behind the PG, medical expert involvement, content alignment with common sense, and feedback from trusted sources	34.83% (62/178) patients perceived lack of credibility as a barrier for seeking or reading PG74.06% of patients believe the credibility of PGs depends on the institutions responsible for its development. 59.43% of patients believe the involvement of medical experts is key to PG credibility	Both qualitative and quantitative findings converge on the importance of credibility in patient engagement with PGs. The qualitative data explains why patients value credibility, such as trust in institutions, medical experts, and peer feedback, while the quantitative data highlights the prevalence of credibility concerns. The qualitative findings provide detailed insights into the factors influencing credibility, like familiarity with trusted sources, while the quantitative results quantify the significance of these factors, such as the high importance of institutions and medical experts in establishing credibility	Confirmed and expanded findings
Attractiveness of PGs increases patient engagement and interest in using them	Patients indicated that the presentation of PGs should align with their visual and auditory preferences, including factors such as presentation form, amount of information, and format.	12.36% (22) of patients viewed the misalignment of PG presentation with their visual and auditory preferences as a barrier. 10.03% (32/319) read PGs in purely textual format. 64.89% (207/319) read PGs with illustrated information, with 31.40% (65/207) preferring cartoon illustrations, and 59.80% (122/207) preferring graphics depicting real people. 25.08% (80/319) were indifferent to the presentation style.	Both qualitative and quantitative findings highlight the importance of PG presentation in patient engagement. The qualitative data explains why presentation matters, focusing on visual and auditory preferences, while the quantitative results indicate that a moderate proportion of patients (12.36%) view misalignment with their presentation preferences as a barrier. The qualitative findings offer insights into the specific visual and auditory elements that affect attractiveness, while the quantitative data reveals the prevalence of these preferences, such as the preference for illustrated formats or specific types of graphics	Confirmed and expanded findings
Simplicity of PGs makes them easy to understand and accessible for patients with varying levels of health literacy	Patients emphasized that ease of understanding is crucial for engaging with PGs. They suggested that PGs should be developed in different versions to cater to patients' varying comprehension levels.	12.36% (22/178) viewed difficulty understanding PG information as a barrier to seeking or reading PG or other health information.	Both qualitative and quantitative findings confirm that simplicity affects patient engagement with PGs. The qualitative data provides insights into how PGs can be simplified to address varying comprehension levels, while the quantitative results show that a moderate proportion of patients find difficulty in understanding PG information a barrier	Confirmed and expanded findings
Relevance of PGs ensures the content addresses patients' concerns and provides meaningful health information	Patients indicated that PGs need to provide new knowledge, address their concerns, and demonstrate potential benefits to improve their health or condition.	12.36% (22/178) viewed the perceived lack of new knowledge or the failure of PGs to improve their health as a barrier to reading them.	Both qualitative and quantitative results show that the perceived value of PGs—in terms of providing new knowledge or potential health benefits—affects patient engagement. The qualitative findings explain why this matters, emphasizing the importance of addressing patient concerns and demonstrating health benefits, while the quantitative data highlights the extent to which the lack of perceived value acts as a barrier. The qualitative results offer deeper insights into what makes PGs valuable, while the quantitative data quantifies the prevalence of these concerns.	Confirmed and expanded findings
Timeliness of PGs ensures that the information is up‐to‐date and relevant for patients' current needs	Patients and healthcare professionals emphasized that PGs must be up‐to‐date due to the constant evolution of science and technology, with healthcare professionals highlighting the importance of this factor when recommending PGs.	10.11% (18/178) viewed the outdated nature of PGs as a barrier to reading certain PGs or other health information.	Both qualitative and quantitative findings support the idea that the timeliness of PGs affects patient engagement. The qualitative results highlight why up‐to‐date information is important due to ongoing healthcare advancements, while the quantitative data complements this by indicating that outdated content is perceived as a barrier to engagement by a proportion of patients.	Confirmed and expanded findings
Usability of PGs determines how practical and actionable the information is for patients	Patients desired clear and actionable guidance in PGs, particularly information that they could directly apply to improve their condition without needing additional consultation.	7.30% (13/178) viewed a lack of explicit instructions as a barrier to seeking and reading certain PGs or other health information.	Both qualitative and quantitative findings support the idea that the timeliness of PGs affects patient engagement. The qualitative findings provide a deeper understanding of why timeliness is crucial, particularly with the rapid advancements in science and technology, while the quantitative results confirm that a portion of patients view outdated PGs as a barrier to engagement	Confirmed and expanded findings

Some patients believed that they were not professionals and should leave the decision‐making to clinicians. They indicated that relying on PGs for independent decision‐making would not only increase their mental burden but also create uncertainty regarding their choices.

You should not make decisions on your own about what to do. This includes medication dosage and treatment duration. As a patient, it is not your role to judge your own condition. You are not a medical professional. Only specialized doctors have the authority to assess and determine the treatment for your condition. They will inform you about the medication you should take, how to take it, and when to come back for follow‐up appointments. It is crucial to follow the doctor's instructions and not make decisions independently, thinking that you are fine and don't need further check‐ups.

[Patient D]

Patients always valued clear explanations about the recommendations provided in the PGs to help them make decisions, particularly when it came to crucial medical decisions. It was important to comprehensively detail the treatment options, associated benefits and risks, and the factors considered in formulating these recommendations.

Nowadays, when the doctor gives you a treatment option, they usually prefer to tell you how good it is. I know that the doctor must have chosen the best treatment option for me, but I would actually like to know what are the harms of this option, and whether it is something I can handle.

[Patient N]

The clinicians indicated that PGs, which included enough information to assist patients in decision‐making, could better assist them in communicating with patients about certain medical decision‐making issues.

I think if the patients come in and you realize that he's read the relevant guidelines and it's easier for you to communicate with patients. So if each patient has a copy of the patient guideline beforehand, it's probably better in terms of reducing communication costs for healthcare professionals and improving treatment outcomes for patients.

[Endocrinologist X]

Quantitatively, only 32.25% of patients said that they needed a PG to assist them in decision‐making independently, 28.75% expressed the desire for a PG that provides sufficient information to support informed decision‐making, yet they preferred not to make decisions independently (see Appendix).

#### 3.2.3 Key Attributes of PG Influencing Patients' Use or Adoption of PGs

1. Qualitative findings

**Figure 1:**
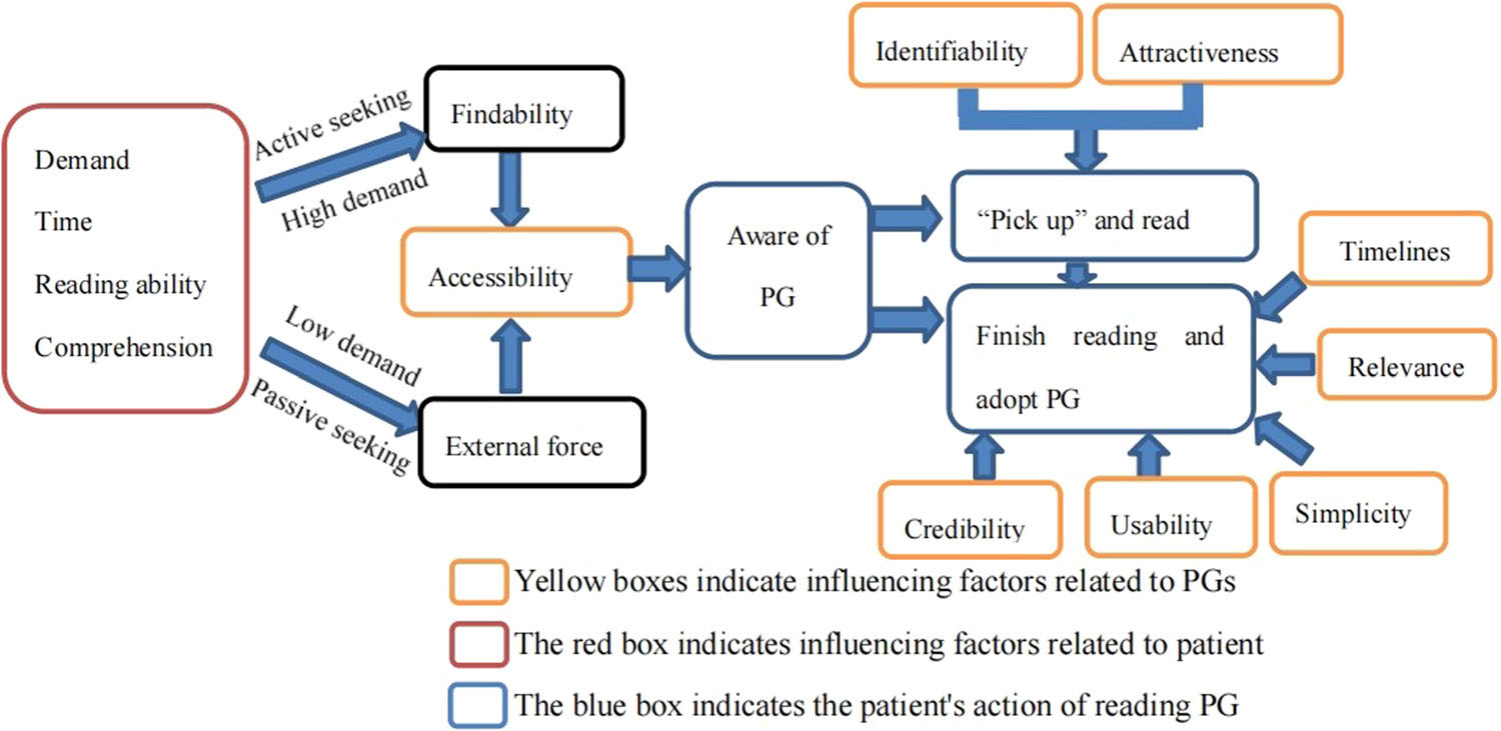
Patients' decision‐making pathways for accessing and reading PGs (based on interview findings). PG, patient guideline.

We identified 8 key attributes of PGs that resonate with patients and influence their interaction with these PGs. These attributes are critical at different stages of patient engagement with PGs. Through narrative exploration, we mapped out patients' decision‐making processes regarding the use or adoption of PGs, which are visually represented in Figure 1. This figure highlights the journey patients take, from recognizing a need to fully engaging with a PG, while illustrating the dynamic interaction between these attributes and patient behaviours at various stages. Figure 1 is based on qualitative interview data.

At the onset, patients' intrinsic needs emerged as the primary impetus for PG exploration. Upon identifying a need, patients would assess their own capacity to engage, considering factors such as literacy and time availability. This internal evaluation dictated whether patients embark on an active search for PGs or settled for passive engagement. Active seekers prioritized the findability of PGs, preferring easily obtainable resources that align with their needs. Conversely, passive readers relied on external prompts, such as recommendations from healthcare providers, to direct their attention towards PGs.

The decision to engage further hinges on the identifiability and attractiveness of PG. Patients were more inclined to delve into PGs provided by trusted sources, whereas those from less reputable origins might be overlooked. Subsequent commitment to reading and implementing PG recommendations was contingent upon the perceived credibility, usability and timeliness, relevance, and simplicity of the PG.

2. Quantitative findings

In the survey, we found the most frequently identified PG‐related reason for not seeking and reading PGs was ‘lack of credibility of information in PGs,’ accounting for 34.83%. Other common reasons identified were ‘Difficulty finding PGs’ (24.16%) and ‘Difficulty immediately recognizing the relevance of PGs’ (21.35%).

When surveyed about their preferred sources for obtaining PGs, medical staff emerged as the most popular choice, accounting for 65.79% of responses. Followed by ‘books and magazines’ (42.11%) and the official WeChat accounts or websites of hospitals or other professional institutions (34.21%).

When asked about how they judged the credibility of the health‐related information they read, 74.06% chose institutions responsible for developing the information, 59.43% chose medical experts who involved in the development of the information.

The survey showed that most patients (59.80%) preferred graphics depicting real people and 31.40% preferred cartoon illustrations (see Appendix).

### 3.3 Mixed Methods Findings

This mixed‐methods study revealed three findings that were confirmed through both qualitative and quantitative data: limited understanding of PGs, a preference for personalized recommendations, and the influence of recommendation strength on decision‐making. Two findings related to patients' attitudes towards and understanding of PGs were confirmed and expanded by the integration of both data types, including heterogeneous attitudes towards PGs and diverse preferences regarding their use in decision‐making. The qualitative data provided rich insights into patient perspectives, while the quantitative data quantified these insights, offering a more comprehensive understanding of patient views on PGs (see Table 1).

The mixed‐methods results also demonstrated both confirmatory and expansive relationships across eight key attributes influencing patients' use or adoption of PGs. Quantitative data validated the presence of these factors, showing that they affect a moderate proportion of patients (typically 10%–30%). Qualitative findings, in turn, provided complementary insights by delving into the reasons behind these factors and suggesting practical solutions to address them, such as tailoring content, improving categorization, and ensuring up‐to‐date information.

## 4 Discussion and Conclusion

### 4.1 Discussion

One of the most critical findings of this study, is the widespread lack of awareness and accurate understanding of the role and purpose of PGs, among patients. As revealed in our study only 26.5% of patients had heard of PGs, and among those, 71.7% perceived PGs as mere health education materials or general guidance. This situation reflects a challenge observed in some international studies, where patient awareness of PGs is generally low, with notable variation across different countries. For instance, Blödt et al. found that 45% of cancer patients in Germany were aware of PGs, but there is still room for improvement in their perception and use [33]. Similarly, Liu et al. surveyed 1319 respondents in China, including patients, clinicians and guideline developers, and found that only 18.3% of all respondents were familiar with or had participated in the development of PGs. Among patients specifically, awareness was low, reflecting the widespread gap in understanding PGs [34]. The low awareness among patients can be attributed to several factors [14]. First, the promotion of PGs to patients is often insufficient. This means patients may not be effectively informed about the existence and benefits of these PGs. Another significant factor is the perception that PGs are primarily for health professionals and have little or no relevance for patients or the public. This perception can lead to a lack of awareness and engagement with PGs. In China, the relatively lower awareness of PGs among patients is also influenced by specific cultural factors. Chinese patients exhibit a significant reliance on healthcare providers, with 37.75% never seeking health information outside of their healthcare provider, reflecting a strong trust in direct medical advice. This trust may limit the recognition and use of supplementary resources like PGs. Additionally, 65.79% of patients expressed that they would be willing to read PGs if these were provided or recommended by their doctors, highlighting the emphasis on traditional medical authority [21]. To address the low awareness of PGs and considering the specific cultural factors in China, it is recommended to enhance the promotion and education of PGs within healthcare institutions, ensuring that doctors actively introduce the role and access of PGs to improve patient awareness. Moreover, effectively integrate PGs into the healthcare service process so that doctors recommend relevant PGs during treatment advice, meeting patients' needs for authoritative guidance.

Our research uncovers a significant insight from patients regarding the recommendation for patients in PGs. This aligns with previous research [14], which indicates that many patients express concerns about the PGs' general nature not aligning with their specific circumstances. However, our study goes further to identify a potential solution to this concern. Patients expressed the necessity for PGs to acknowledge individual differences and provide recommendations that are more tailored based on individual factors like age, co‐morbidities and weight, ensuring that they address patients' specific needs and support informed decision‐making. Our study not only corroborated this phenomenon but also revealed that 60.25% of patients expressed a need for general recommendations from PGs. This suggests that, within the Chinese healthcare context, while personalized recommendations are important, general recommendations remain valuable. Considering the unique cultural factors in China, we gain a deeper understanding of why patients seek general PG recommendations. First, there are significant disparities in medical resources and service quality between urban and rural areas in China [35, 36]. Major cities have abundant medical resources, whereas remote areas often face inadequate medical conditions. This uneven distribution can lead to inconsistent advice from doctors or insufficient medical information. In such cases, general PG recommendations offer consistency and standardized reference points, helping patients make informed decisions amidst resource disparities. Second, the demand for personalization may be influenced by the long‐standing impact of ‘differentiated diagnosis and treatment’ in Traditional Chinese Medicine (TCM) within Chinese culture [37], where many patients seek individualized recommendations.

In this study, many patients misunderstood the strength of recommendations in PG, particularly interpreting a ‘strong recommendation’ as indicating that their condition is more severe or requires urgent treatment. This misunderstanding may be influenced by cultural factors. In Chinese culture, strong language is often associated with severity or urgency, leading patients to misinterpret the strength of recommendation as a reflection of the seriousness of their condition rather than the strength of supporting evidence. To avoid such confusion, it is essential that explanations of recommendation strength are culturally sensitive, making it clear that the strength of the recommendation does not equate to the urgency or severity of the disease. In fact, the study reveals that 69.75% of patients found the strength of recommendations helpful when the reasons behind it were properly explained, rather than relying on symbols or technical terms. This aligns with Fearns et al.'s recommendation [38], which emphasizes the importance of using accessible language to communicate the strength of recommendations, helping patients make more informed decisions.

The characteristics of a ‘good’ PG, as identified by patients in this study, included accessibility, identifiability, attractiveness, credibility, usability, timeliness, relevance, and simplicity, align with the honeycomb user experience model [39], which was used to test PGs as a framework [15]. These attributes reflect the importance of user‐friendly design, reliable sources, clear organization, and visually appealing presentation to enhance patient engagement and compliance. Despite these essential characteristics, many PGs in China may lack user testing [8, 24], a crucial step to ensure PGs meet the needs and expectations of patients. This oversight highlights the importance of incorporating user testing into the development process of PGs in China to enhance their usability and impact on patient care.

One of the eight attributes, timeliness, deserves special attention. Keeping PGs up‐to‐date with the latest research is crucial for their acceptance by patients and recognition by clinical staff. However, in China, there has been a notable lack of updates to PGs, even those developed over 5 years ago [24]. This situation underscores the urgent need to establish specialized organizations in China, dedicated to the development and regular updating of PGs. These organizations would mirror successful models in other healthcare systems, such as NICE and SIGN, ensuring that PGs remain current and relevant to both patients and healthcare professionals.

While our study provides valuable insights, several limitations should be considered. The focus on adult patients with chronic diseases may not capture perspectives from acute or paediatric populations, and the small number of certain participants, such as only one GP, limits the depth and representativeness of clinician perspectives. Restricting the study to first‐tier cities raises concerns about generalizability across different economic contexts in China, as efforts to include rural patients may not fully reflect economically disadvantaged areas. Additionally, the methodology, where patients were introduced to PGs during the study, may influence their perspectives, introducing potential biases. Despite these limitations, our study offers valuable insights into the development and implementation of PGs in China.

Furthermore, the relatively low prevalence rates (20%–30%) observed in the quantitative findings for key attributes identified in the qualitative research suggest potential methodological limitations: qualitative approaches provide rich, open‐ended insights into patient attitudes, while quantitative methods rely on closed‐ended questions, which may limit the expression of nuanced patient perspectives. Cultural factors, such as patients' reliance on traditional medical practices and sample heterogeneity may also influence these results.

### 4.2 Conclusion and Practice Implications

This study highlights the cultural factors shaping Chinese patients' awareness and attitudes towards PGs. Despite their potential benefits, challenges such as low awareness and misconceptions persist. To improve PG effectiveness, it is essential to promote them more widely in healthcare settings and clearly communicate their practical benefits. PGs should balance personalized care with standardized guidance, addressing both individual patient needs and the broader healthcare context, considering China's cultural preference for individualized care and disparities in medical resources. Establishing specialized PG development organizations or collaborating with existing CPG bodies is key to ensuring PGs remain up‐to‐date. Additionally, integrating user feedback before release will help align PGs more closely with patient needs and enhance their impact.

## Author Contributions

**Lijiao Yan:** conceptualization, methodology. **Ning Liang:** methodology, data curation. **Zeyu Yu:** investigation, data curation. **Loraine Cook:** writing–review and editing, writing–original draft. **Sarah E. Scott:** writing–review and editing. **Karen Graham:** writing–review and editing. **Xing Liao:** writing–review and editing, validation. **Haili Zhang:** visualization, validation. **Jiale Hu:** methodology, validation, writing–review and editing, conceptualization. **Nannan Shi:** writing–review and editing, funding acquisition, conceptualization. **Jianping Liu:** writing–review and editing, project administration, methodology, conceptualization. The authors achieved investigator triangulation and reached a consensus on the findings, and all have agreed on the final version.

## Ethics Statement

Ethical approval was obtained from the Beijing University of Chinese Medicine for conducting this interview (Approval Number: 2022BZYLL0706).

## Consent

All participants received an introduction to this study in advance and gave informed oral consent to participate before the session began. The participants were made aware that they could withdraw from the study at any time, that their information would be kept confidential, and that they would remain anonymous in any publications.

## Conflicts of Interest

Co‐author Karen Graham and Sarah E. Scott are authors for GIN Public Tookit, which is cited in this manuscript. Other authors declare that the research was conducted in the absence of any commercial or financial relationships that could be construed as a potential conflicts of interest.

## Supporting information

Supporting information.

## Data Availability

The datasets used and/or analysed during the current study available from the corresponding author on reasonable request.
