# Supplementary material for: Perceptions and Attitudes of People With Cancer and Diabetes Towards Patient Guidelines: A Mixed Methods Study
Source: Health Expect. 2025 Jan 28;28(1):e70164. doi: 10.1111/hex.70164 (PMC11775387; doi:10.1111/hex.70164)
Supplement: Supplementary file 1 — Supporting information. [file HEX-28-e70164-s001.doc]

Table A1 A detailed comparison of patient-directed knowledge tools

| **Tool Type** | **Purpose** | **Scope** |
| --- | --- | --- |
| **PEMs** | To inform or educate patients by providing foundational information about diseases, treatments, and health management | Helps patients understand their health conditions without providing specific treatment recommendations |
| **PVGs** | Simplified, patient-friendly versions of CPGs that summarize treatment options, benefits, and risks | Assists patients in understanding treatment options and making informed decisions based on CPG recommendations |
| **PDAs** | To support decision-making by presenting the pros, cons, risks, and benefits of treatment options | Helps patients evaluate treatment choices based on their personal values and preferences |

PVG: patient version of guideline; PEM:patient education materials; PDA: patient decision aids; CPG: clinical practice guidelines

Table A2 Characteristics of participants from the qualitative interviews

| No. | Age | Gender | Education | Patient/carer | Interview site |
| --- | --- | --- | --- | --- | --- |
| A | 45 | Female | Junior college | Carer of people living with diabetes | Outpatient clinic |
| B | 61 | Male | Postgraduate | People living with diabetes | Outpatient clinic |
| C | 63 | Male | Postgraduate | People living with diabetes | Outpatient clinic |
| D | 45 | Male | High school | People living with diabetes | Outpatient clinic |
| E | 53 | Male | Secondary school | People living with diabetes | Outpatient clinic |
| F | 38 | Female | Junior college | People living with diabetes | Outpatient clinic |
| G | 58 | Female | Secondary school | People living with diabetes | Outpatient clinic |
| H | 54 | Female | High school | People living with diabetes | Outpatient clinic |
| I | 27 | Female | Postgraduate | People living with diabetes | Online |
| J | 32 | Male | Undergraduate | People living with diabetes | Online |
| K | 64 | Male | Undergraduate | People living with diabetes | Online |
| L | 42 | Female | Undergraduate | Cancer survivors | wardroom |
| M | 58 | Male | Secondary school | Cancer survivors | wardroom |
| N | 62 | Male | Undergraduate | Cancer survivors | wardroom |
| O | 45 | Male | High school | Cancer survivors | wardroom |
| P | 54 | Female | High school | Cancer survivors | wardroom |
| Q | 37 | Female | Graduate | Oncologist | Online |
| R | 34 | Female | Graduate | Oncologist | Online |
| S | 36 | Female | Undergraduate | Oncology Nurse | Office |
| T | 26 | Female | Junior college | Oncology Nurse | Office |
| U | 36 | Female | Graduate | Head Nurse, Endocrinology Department | Online |
| V | 30 | Female | Graduate | Endocrine nurse | Online |
| W | 34 | Female | Graduate | Community general practitioners | Online |
| X | 36 | Female | Undergraduate | Endocrinologist | Outpatient meeting room |
| Y | 38 | Female | Postgraduate | Endocrinologist | Online |

Table A3 Characteristics of survey population

| Variables | Categories | n | % |
| --- | --- | --- | --- |
| Type of patient | Cancer survivors | 137 | 34.25 |
|  | People living with diabetes | 263 | 65.75 |
| Gender | Male | 213 | 53.25 |
|  | Female | 187 | 46.75 |
| Age | 15-20 | 5 | 1.25 |
|  | 20-30 | 17 | 4.25 |
|  | 30-40 | 58 | 14.50 |
|  | 40-50 | 83 | 20.75 |
|  | 50-60 | 105 | 26.25 |
|  | 60-70 | 89 | 22.25 |
|  | >70 | 43 | 10.75 |
| Educational level | Primary school and below | 45 | 11.25 |
|  | Secondary school | 111 | 27.75 |
|  | High school | 79 | 19.75 |
|  | Junior college | 72 | 18.00 |
|  | Undergraduate or over | 93 | 23.25 |
| Type of residence | Urban | 342 | 85.50 |
|  | Rural | 58 | 14.50 |
| Characteristics of cancer survivors | | | |
| Staging of tumors | Early stage | 29 | 21.17 |
|  | Mid stage | 32 | 23.36 |
|  | Advanced stage | 46 | 33.58 |
|  | Not known | 30 | 21.90 |
| Treatment | Chemotherapy | 82 | 59.85 |
|  | Radiotherapy | 53 | 38.69 |
|  | Surgery | 38 | 27.74 |
|  | Other treatment | 63 | 45.99 |
| Characteristics of people living with diabetes | | | |
| Complications or not | Yes | 141 | 53.61 |
|  | No | 122 | 46.39 |
| Blood glucose control | basically normal | 70 | 26.62 |
|  | Occasionally abnormal | 95 | 36.12 |
|  | Frequently irregular | 23 | 8.75 |
|  | Not known | 75 | 28.52 |
| Duration of disease | <1 year | 45 | 17.11 |
|  | 1-3 year | 46 | 17.49 |
|  | 3-5 year | 31 | 11.79 |
|  | >5 year | 141 | 53.61 |
| Use of injectable hypoglycemics | Yes | 141 | 53.61 |
|  | No | 122 | 46.39 |

n: number

Table A4 Themes and subthemes based on qualitative findings

| **Theme** | **Subtheme** | **Description/Example** |
| --- | --- | --- |
| 1 Perception of PGs | 1.1 Limited Understanding and Misunderstanding of PGs | Patients and clinicians have a limited understanding of PGs, often viewing them as:  --General health education materials or medical care guides: often distributed by hospitals or health institutions  --Disease-related informational books: intended for the general public,which were usually available for purchase in bookstores or online |
| 2 Attitudes toward PGs | 2.1 Attitudes toward PGs are heterogeneous | Attitudes toward PGs varied, with some viewing them as scientific, credible, and patient-centered tools, while others felt PGs were not helpful and that information from healthcare professionals was sufficient |
|  | 2.2 The value of PG recommendations is recognized, but more personalized advice is desired | PG recommendations are seen as credible and empowering for informed decision-making. But there is concern about their individual applicability, tailored recommendations based on specific health needs are preferred |
|  | 2.3 Strength of recommendations influences decision-making for some patients, while others find it unhelpful | Patients expressed varying views on the strength of recommendations in PGs. Some found the strength of recommendations valuable, as it helped them make more informed decisions based on scientific evidence rather than personal preferences. Others felt the strength was irrelevant or confusing, often misunderstanding it as an indicator of illness severity or treatment urgency. Additionally, some participants felt that clearer explanations of the strength of recommendations helped them feel more confident in their choices, while others found icons used to convey strength difficult to interpret |
|  | 2.4 Patients have varying preferences regarding the use of PGs for decision-making | Patients have different preferences when it comes to using PGs for decision-making. Some prefer clinician-led decision-making, feeling that PGs add unnecessary mental burden. However, others find the clear information and detailed explanations provided by PGs helpful in making informed decisions. PGs are also seen as valuable in enhancing communication between patients and clinicians, reducing misunderstandings, and improving treatment outcomes. |
| 3 Key attributes of PG influencing patients’ use or adoption of PGs | 3.1 Accessibility | Patients value easy access to PGs and prefer sources that are readily available and easily navigable.  - Findability of PGs is crucial: High-demand patients expect easy access to PGs, while low-demand patients may prefer to receive them from hospital during the visit, by the community etc.  - Preference for direct accessibility: Patients prefer PGs that can be easily accessed on mobile devices without needing to search or navigate through other platforms. For example, Patient W commented, "Doctors might say, 'This is good, you can take a look,' but I would rather use something that’s readily available on my phone. I don’t like having to search for a WeChat public account, it’s just too much trouble." |
|  | 3.2 Identifiability | PGs should be easily recognizable through attention-grabbing presentation formats, clear covers, and titles. Extensive content should be categorized clearly, and including a table of contents improves accessibility.Cear frameworks, such as STAR or SCQA, would help them quickly identify the most pertinent information |
|  | 3.3 Attractiveness | PGs should align with patients' visual and auditory preferences to increase engagement and usability  Example:Patient A mentioned: "For me, as soon as I see the pictures, I know what to do, I don't need to read the words. I prefer guidelines like that." |
|  | 3.4 Credibility | PGs should be objective, free from conflicts of interest, and come from trusted sources such as healthcare professionals and reputable organizations. Credible feedback and information enhance trust in the PG content. |
|  | 3.5 Usability | PGs should provide actionable information that patients can implement, with treatments that align with doctors' recommendations and are locally accessible.  Example: Patient F noted: "There’s a difference between theory and reality. The guide may recommend precise quantities for ingredients, but in daily life, it's difficult to follow such specifics. We just want to know if we can eat dishes like broccoli and shrimp, without needing to measure everything down to the gram." |
|  | 3.6 Timeliness | PGs should be based on the latest research findings to ensure they reflect current evidence and best practices |
|  | 3.7 Relevance | PGs should address patients' concerns, provide new knowledge, and offer intervention-oriented information that is directly relevant to patients' lives and situations. This ensures the PG can make a real difference in their health management  Example: Patient I mentioned, “I pay closer attention to diabetic foot care because although I know about it, I haven't learned much. Since it relates to me, I’ll read it more carefully.” |
|  | 3.8 Simplicity | PGs should be tailored to different patient levels, avoid overly technical language, and use large fonts for better readability  Example: Patient I highlighted that complex language can be overwhelming for non-expert readers: "The guidelines are very professional, especially the last one on type 2 diabetes management. If you give it to regular patients, they might not have the patience to read it all." |

PG:patient guideline

Table A5 Results of the survey on patients' perception, attitudes and the perceived PG-related factors influencing their use or adoption of PGs

| **Questions** | **Options** | **n (%)** |
| --- | --- | --- |
| **Perception of PGs** | | |
| **Have you heard of PGs?** | Yes | 106 (26.50) |
|  | No | 294 (73.50) |
| **What is PG as you understand?** (For patients who have heard of PGs) | General health education materials or as medical care guides | 76 (71.70) |
|  | Disease-related informational books intended for the general public | 17 (16.04) |
|  | Guidelines posted on guideline websites, such as Medical Pulse, Chinese Medical Association website, etc. | 16 (15.09) |
| **Attitudes Toward PGs** | | |
| Except for obtaining disease-related knowledge from healthcare providers, are you willing to seek any other source of health-related information like PGs? | Yes | 292 (73.00) |
|  | No | 108 (27.00) |
| **Attitude Toward Recommendations for PGs** | | |
| **Which answers will you choose if you have a decision-making issue?** | Doctor's advice | 303（75.75） |
|  | Recommendations from a group of experts based on global research evidence | 241（60.25） |
|  | Your own unique experiences | 159（39.75） |
|  | Advice from others in similar situations | 175（43.75） |
|  | Suggestions given in other ways | 73（18.25） |
| **When making decisions about your health, which type of recommendation do you think would be most helpful?** | Recommendations from experts based on general scientific knowledge, applicable to most patients | 36 (14.94) |
|  | Recommendations tailored to my specific health condition | 48 (19.92) |
|  | Either type of recommendation works for me, as long as it is evidence-based | 24 (9.95) |
|  | Both types of recommendations are needed | 133 (55.19) |
| **Attitude Toward Strength of Recommendations** | | |
| **Does the strength of recommendations affect your adoption of that recommendation?** | The strength of recommendation doesn't affect me | 92 (23.00) |
|  | The strength of recommendation has an impact on me | 163 (40.75) |
|  | The strength of recommendation has an effect on me, and I should go for only strongly recommended suggestions | 116 (29.00) |
|  | I'm not sure | 29 (7.25) |
| **Attitude Toward PG Assistance in Decision-Making** | | |
| **Do you need a PG to assist you in making disease-related decisions without the support from healthcare providers?** | I need PGs to help me make decisions on my own | 129 (32.25) |
|  | I need PGs enough information to help, but don't want to make decisions by myself | 115 (28.75) |
|  | I don't need a PG to help me make decisions, I go straight to the doctor | 100 (25.00) |
|  | I am not sure | 56 (14.00) |
| **The perceived PG-related factors influencing their use or adoption of PGs** | | |
| **What PG-related factors do you perceive that will hinder your seeking for or reading PGs (n=178)?** | Difficulty finding PGs | 43 (24.16) |
|  | Difficulty recognizing the relevance of PGs | 38 (21.35) |
|  | PGs offer no new information | 22 (12.36) |
|  | Lack of credibility of information in PGs | 62 (34.83) |
|  | Lack of explicit instruction of information in PGs | 13 (7.30) |
|  | The information is outdated | 18 (10.11) |
|  | The presentation of PG does not align with the patient's visual and auditory preferences | 22 (12.36) |
|  | The information is difficult to understand | 22 (12.36) |
| **Preferred and Trusted Sources of PGs** | | |
| **Which one is your preferred and trusted source of PG?** | Medical staff | 210 (65.79) |
|  | Books, magazines, etc. | 135 (42.11) |
|  | Hospital or professional institution's official WeChat account or website | 109 (34.21) |
|  | Search engines like Baidu | 67 (21.05) |
|  | TV, radio, etc. | 26 (7.89) |
|  | Healthcare-related websites for seeking medical advice | 17 (5.26) |
|  | Patient support group | 17 (5.26) |
|  | Other sources (e.g., TikTok) | 26 (7.89) |
| **Credibility of Information** | | |
| **What information did you rely on to judge the credibility of the information?** | Institutions responsible for developing the information | 184 (74.06) |
|  | The medical experts involved in the development of the information | 148 (59.43) |
|  | The process of developing the information material | 75 (30.19) |
|  | Contact information for relevant sources | 60 (24.06) |
| **Preferred Presentation Style** | | |
| **Which presentation style of health-related information would you prefer?** | Text-only | 32 (10.03) |
|  | Illustrated | 207 (64.89) |
| **Which of the following graphic styles do you prefer?** | Doesn't matter | 80 (25.08) |
|  | Cartoon illustrations | 65 (31.40) |
|  | Graphics depicting real people | 122 (59.80) |

**Material and methods**

**The objectives of this study are:**

Qualitative Objectives:

- To explore how patients view PGs (RQ1).
- To understand the reasons behind these attitudes (RQ2).
- To identify specific factors related to PG content, design, presentation, and management that may influence patients' use or adoption of PGs (RQ3).

Quantitative Objectives:

- To measure the level of patient awareness of PGs (RQ4).
- To assess the proportion of patients with positive or negative attitudes towards PGs (RQ5).
- To quantify how frequently different PG-related factors are reported that influence their use or adoption of PGs (RQ6).

We applied an exploratory sequential mixed methods design, as it allows exploration of a phenomenon from participants' perspective and to develop a questionnaire that “based on the culture and setting of the research participants rather than pulled off the shelf for use”[[1]](#endnote-2). Firstly, semi-structured interviews with open-ended questions were conducted to collect data from the perspectives of multiple stakeholders, including patients, physicians, and nurses. The initial qualitative phase was used because: a) following a scoping literature review we were unable to find a questionnaire to determine patients’ perceptions and attitudes towards PG; b)patients’ perceptions and attitudes toward PG are contextual and may be influenced by local socioeconomic status and medical environment. Based on the qualitative analysis, a structured questionnaire was developed. Finally, a subsequent quantitative study was conducted to investigate and quantify patients’ perception, attitudes, and PG-related factors influencing their use or adoption of PGs among a larger sample of patients. We followed the Good Reporting of A Mixed Methods Study (GRAMMS)[[2]](#endnote-3) and the Consolidated Criteria for Reporting Qualitative Research (COREQ)[[3]](#endnote-4).

**1 Sample and setting**

The study was conducted in 4 hospitals in two cities, Fangshan Hospital of Traditional Chinese Medicine (Beijing), Tsinghua Changgong Hospital (Beijing), and Peking University First Hospital (Beijing), and the University of HongKong Shenzhen Hospital (Shenzhen).

This study incorporated both patient and clinician perspectives during the interview phase. The rationale for integrating patient and clinician data sources in our study was to achieve a comprehensive understanding of the diverse views and experiences related to PGs. While patient perspectives are vital, they may not encompass the full spectrum of diverse views and experiences related to PGs due to individual differences, knowledge limitations, and challenges in expressing their needs. Clinicians, who interact with various patients, can provide valuable insights into common views and experiences. This integration helps us develop more effective PGs that can be applied in real-world clinical settings, benefiting both patients and clinicians.

The inclusion criteria for patients were: (1) patients who came to the clinic due to high blood glucose (pre-diabetes, diabetes mellitus, and complications stage) and oncology; (2) age>18 years old; and (3) able to provide informed consent. The inclusion criteria for clinician were (1) registered nurses or registered physicians with a bachelor's degree or higher, and (2) at least 5 years of experience in endocrinology or oncology.

Purposive sampling was employed during qualitative and quantitative phase. The sampling frame was determined based on key variables,including ages, genders, education levels, disease stages, and living environments (rural or urban). These variables were selected to ensure that the sample included diverse subgroups relevant to our research aims, thus providing a more comprehensive understanding of the various views and experiences associated with PGs. To ensure a balanced distribution across the identified variables, We deliberately selected participants representing these characteristics. Specific quotas were established for each variable based on the demographic profiles of the patient population in the hospitals. During participant recruitment, we monitored the representation of each category to maintain this balance, adjusting our recruitment strategies as necessary to meet the desired distribution.

The sample size for the qualitative study was determined based on the principle of data saturation, where data collection continues until no new insights are gained. For the quantitative survey, being an exploratory study, sample sizes were not calculated but rather determined by including as many patients as possible within the constraints of limited time and financial support.

**2 Data collection**

The qualitative data were collected from May 2022 -August 2022 using an interview guide(qualitative phase Ⅰ). We chose a combination of online and face-to-face interviews depending on the schedules of patients and clinicians. Personal information about gender, age, occupation was asked at the beginning of the interview. For the face-to-face interviews, we chose a quiet room to conduct the interviews and used IFLYREC app (iFLYTEK CO.LTD, Version: 6.0.3171) to record and transcribe. During the online interviews, the Tencent Conference recording function was used directly to record language. All interviews were conducted by the first and third authors, who are doctoral candidates specializing in evidence-based medicine with prior experience in conducting qualitative interviews. They had also received specific training in qualitative research and interview techniques prior to the study. Each interview lasted approximately 45 minutes. We established a professional relationship with the participants prior to the interviews, ensuring transparency about the purpose of the study and our roles as interviewers. Participants were informed about the interviewer's background and expertise in the relevant field.

The quantitative data were collected from August 2022-November 2022 using an interviewer-administered questionnaire. The questionnaires were administered by trained interviewers who guided participants through the questions to ensure they fully understood them without influencing their responses. This process was facilitated through the WenJuanXing online survey service (English name “SurveyStar”, Changsha Ranxing Science and Technology, Shanghai, China), which is an online survey platform akin to SurveyMonkey. Patients were prompted to scan the QR code of the questionnaire in WenJuanXing and complete it using their cell phones.To minimize interviewer bias, all interviewers underwent specific training to maintain neutrality, adhere strictly to a standardized script, and avoid leading questions. Participants were also informed that their responses were anonymous and that they could withdraw from the study at any time. These measures were implemented to ensure that participants’ responses were genuine and free from undue influence.

For outpatients who met the inclusion criteria and agreed to participate, physicians were responsible for recommending them to the researcher for interviews. This approach was chosen because physicians had direct and detailed knowledge of their patients' health conditions, making them well-placed to identify individuals who would be most relevant for the study based on their medical history and current health status. After being recommended by their physician, the researcher then explained the purpose and significance of the study to the patients and obtained their signed informed consent. For inpatients in the ward, nurses facilitated the process by introducing the researcher to patients, asking if they were willing to participate, and interviews were conducted with those who consented. For the recruitment of clinician, we initially identified a key individual in each hospital, who then recommended eligible participants for the interviews.

**3 Data collection instruments**

3.1 Interview guide

The integration of the Technology Acceptance Model (TAM) [[4]](#endnote-5) and the conceptual framework of PGs[[5]](#endnote-6) guided the development of the interview guide in a comprehensive manner. TAM provided a foundation to explore patients' acceptance of PGs by focusing on perceived usefulness and perceived ease of use. It serves as a foundational tool to understand patients' acceptance of PGs, specifically regarding their attitudes and behavioral intentions, which aligns with our qualitative objectives to explore how patients view PGs (RQ1) and understand the reasons behind these attitudes (RQ2). However, we recognize that the TAM's application in our study is somewhat limited. The interview guide encompasses broader themes beyond just these constructs, allowing for a more comprehensive exploration of patients' views on PGs. The conceptual framework of PGs facilitates a deeper dive into patients' attitudes towards specific core features of PGs, such as health education, evidence-based recommendations, and decision support. This dual approach ensured that we captured both general attitudes towards PGs and more detailed perceptions of their key characteristics. Additionally, insights from preliminary interviews with PG developers were incorporated to ensure relevance and comprehensiveness. See additional file for detailed interview guide.

To ensure the effectiveness of the interview guide, one PG developer, three clinicians, and two patients were invited to review and provide feedback, resulting in necessary revisions. The initial two interviews served as a pretest to assess language appropriateness and question effectiveness. Questions with unsatisfactory responses were adjusted, and clarifications were incorporated.

**Interview guide for patients**

（1）Perception of PG(patient guideline).

- Where do you typically seek information about your illness?
- Are you aware of diabetes guidelines designed to assist healthcare professionals? Would you be interested in learning more about these guidelines?
- What is your understanding of guideline?

（2）Attitudes Towards PGs

Explain what a guideline and PG is and introduce the concept verbally or through an animation <https://www.bilibili.com/video/BV11t4y1b7pR/)>. Patient Guidelines (PGs) are developed by a team of experts from various fields, who review the best available research to provide clear, evidence-based recommendations to help patients make informed decisions when facing medical choices. Unlike general patient education materials, which typically provide broad information about health conditions or treatments, PGs focus specifically on healthcare decisions that are relevant to the patient's needs. For example: A patient with diabetes might need to decide whether to start insulin therapy, use oral medication, or adopt lifestyle changes. A PG would present the options, their benefits, risks, and the evidence supporting each choice.

Participants were then presented with four existing PGs (diabetic foot, oncology, gout, and pediatric cough) to obtain their feedback on their comprehension, the usefulness of the information, and their overall experience with the existing PGs. The selection of these PGs was based on their relevance to a wide range of patients and the availability of these PGs. Diabetic foot and oncology were directly relevant to our diabetic and oncology cohort, while gout and pediatric cough were chosen to represent conditions common in the general population. This approach was intended to elicit a diverse range of responses and insights into the use of PGs.

**Overall Attitude**

- What is your overall attitude towards using PGs in your healthcare?
- Would you recommend PGs to other patients? Why or why not?
- What potential impact do you think PGs could have on your health management?

**Attitude towards the Role of PGs**

- Do you understand what PGs are supposed to achieve? (Education, decision assistance, collaborative decision-making with your doctor)
- How do you think PGs could better assist you? If education is the primary focus, inquire about preference for how recommendations are presented. If decision assistance is mentioned, ask about desired information to aid decision-making.
- Do you intend to continue using PGs in the future?

**Attitude toward recommendations provided in the PGs**

- How useful do you think the recommendations provided in the PGs would be in helping you make healthcare decisions (e.g., managing blood glucose levels)? Provide an example recommendation.
- How do you interpret strong and weak recommendations in guidelines? (Present a figure illustrating recommendation strength)
- How do you think information like evidence level and recommendation strength should be presented? Provide examples from national and international sources.

（3）Perceived PG-related factors influencing their engagement with PGs

- What factors would influence your decision to continue or stop using PGs?
- How easy do you think it would be to understand and use the information provided in the PGs?
- What challenges, if any, do you anticipate encountering while using PGs?

**Interview guide for health providers**

1. Are you familiar with PGs? If so, how do you define PG? If not, provide an explanation of PGs.
2. How do you perceive the usefulness of PGs for your patients?
3. Can you provide examples where PGs have aided in patient care?
4. How easy do you find it to guide patients in using PGs?
5. What barriers have you noticed patients facing while using PGs?
6. How do you suggest PGs should present recommendations? (Allow providers to share thoughts before presenting Recommendations Presentation Framework)
7. How should information like evidence level and recommendation strength be presented in PGs? (Offer examples from national and international contexts)
8. What is your overall attitude towards incorporating PGs into patient care?
9. Would you recommend PGs to your patients? Why or why not?
10. What is your opinion on the evidence-based recommendations within PGs?
11. How do you assess the decision support features provided by PGs?
12. In what scenarios do you foresee using a PG?

3.2 Questionnaire

We employed building integration techniques[[6]](#endnote-7) to develop the survey questionnaire. Firstly, questionnaire items were formulated based on the themes identified in the interviews. Secondly, the interview findings informed the phrasing of questions and the design of answer options. For instance, in addition to assessing patients' awareness of PGs (question 1), we also inquired about their understanding of PGs as perceived by them (question 2), aligning with interview insights.

This study was exploratory in nature, aiming to explore patients' perceptions and attitudes towards PGs. In exploratory research, the focus is often on data collection and initial analysis, with more in-depth analysis and validation planned for subsequent research phases. Therefore, we prioritized data collection and preliminary analysis over complex reliability and validity testing at this stage. Despite the absence of reliability and validity data, we implemented practical measures to mitigate this limitation. Specifically, the questionnaire items were developed based on interview themes and underwent rigorous pre-testing and expert evaluation. These steps were aimed at enhancing participant comprehension and improving response quality.

The questionnaire underwent pre-testing among 20 patients, using a Think-Aloud[[7]](#endnote-8) method, which resulted in substantial revisions to enhance participants' understanding of several questions. Subsequently, it was evaluated by experts in oncology, diabetes, and questionnaire design from hospitals and universities. A key challenge identified during both the patient pretest and expert feedback stages was the limited understanding of PGs among patients, leading to suboptimal responses. To address this issue, explanations for these concepts were incorporated into the questionnaire, and scenarios were provided to aid patient comprehension and response accuracy. Additionally, experts recommended the inclusion of open-ended questions at the end of the questionnaire to facilitate a broader and deeper exploration of participants' attitudes towards the content, presentation, and dissemination of PGs.

**4 Data Analysis**

The raw data of interviews were first recorded and then transcribed verbatim using the IFLYREC app (iFLYTEK CO.LTD, Version: 6.0.3171). All interview notes and audio recordings were reviewed by both interviewers together on the same day of the interview. We used directed content analysis approaches, as described by Hsieh and Shannon (2005)[[8]](#endnote-9) to analyze the data collected through interview and open-ended questions in the questionnaire. A coding guideline was created collaboratively by the research team, which included the first author, a qualitative researcher, and the second author, a research assistant, to ensure consistency in coding The transcripts were coded using NVivo (V.12 for Microsoft). Data were structured according to a predefined category system. We developed main categories deductively based on the core topics. During the analysis of the material, we inductively complemented and refined the scheme with further categories or subcategories. The final findings were further discussed by the research team, including the first, second, third authors, and the corresponding author, to achieve investigator triangulation and reach a consensus on the findings..

Descriptive statistics were calculated for the demographic variables. Frequencies and percentages were obtained for the individual items of the questionnaire.

The quantitative and qualitative data were integrated using the weaving approachError: Reference source not found and joint display. Weaving approach involves writing both qualitative and quantitative findings together on a theme-by-theme or concept-by-concept basis. While, joint displays ^Error: Reference source not found^ integrates the data by bringing the data together through a visual means to draw out new insights beyond the information gained from the separate quantitative and qualitative results.

1. Creswell, J.W., Plano-Clark, V.L., 2018. Designing and Conducting Mixed Methods Research. 3rd ed. Sage, Los Angeles, CA. [↑](#endnote-ref-2)
2. O'Cathain, A., Murphy, E., & Nicholl, J. (2008). The quality of mixed methods studies in health services research. Journal of health services research & policy, 13(2), 92–98. https://doi.org/10.1258/jhsrp.2007.007074 [↑](#endnote-ref-3)
3. Tong A, Sainsbury P, Craig J. Consolidated criteria for reporting qualitative research (COREQ): a 32-item checklist for interviews and focus groups. Int J Qual Health Care. 2007;19(6):349-357. doi:10.1093/intqhc/mzm042 [↑](#endnote-ref-4)
4. Davis FD. Perceived usefulness, perceived ease of use, and user acceptance of information technology. MIS Q 1989; 13: 319–339.https://doi.org/10.2307/249008 [↑](#endnote-ref-5)
5. Dreesens, D., Stiggelbout, A., Agoritsas, T., Elwyn, G., Flottorp, S., Grimshaw, J., Kremer, L., Santesso, N., Stacey, D., Treweek, S., Armstrong, M., Gagliardi, A., Hill, S., Légaré, F., Ryan, R., Vandvik, P., & van der Weijden, T. (2019). A conceptual framework for patient-directed knowledge tools to support patient-centred care: Results from an evidence-informed consensus meeting. Patient education and counseling, 102(10), 1898–1904. https://doi.org/10.1016/j.pec.2019.05.003 [↑](#endnote-ref-6)
6. Fetters, M. D., Curry, L. A., & Creswell, J. W. (2013). Achieving integration in mixed methods designs-principles and practices. Health services research, 48(6 Pt 2), 2134–2156. https://doi.org/10.1111/1475-6773.12117 [↑](#endnote-ref-7)
7. Banning M. The think aloud approach as an educational tool to develop and assess clinical reasoning in undergraduate students. Nurse Educ Today. 2007;28(1):8–14. [↑](#endnote-ref-8)
8. Hsieh, H. F., & Shannon, S. E. (2005). Three approaches to qualitative content analysis. Qualitative health research, 15(9), 1277–1288. https://doi.org/10.1177/1049732305276687 [↑](#endnote-ref-9)
